# Structural basis of human ORP1-Rab7 interaction for the late-endosome and lysosome targeting

**DOI:** 10.1371/journal.pone.0211724

**Published:** 2019-02-05

**Authors:** Junsen Tong, Lingchen Tan, ChangJu Chun, Young Jun Im

**Affiliations:** College of Pharmacy, Chonnam National University, Gwangju, Republic of Korea; Simon Fraser University, CANADA

## Abstract

Oxysterol-binding protein (OSBP) and OSBP-related proteins (ORPs) constitute a family of lipid transfer proteins conserved in eukaryotes. ORP1 transports cholesterol at the interface between the late endosomes/lysosomes (LELs) and the endoplasmic reticulum (ER). ORP1 is targeted to the endosomal membranes by forming a tripartite complex with the LE GTPase Rab7 and its effector RILP (Rab7-interacting lysosomal protein). Here, we determined the crystal structure of human ORP1 ANK domain in complex with the GTP-bound form of Rab7. ORP1 ANK binds to the helix α3 of Rab7 located away from the switching regions, which makes the interaction independent of the nucleotide-binding state of Rab7. Thus, the effector-interacting switch regions of Rab7 are accessible for RILP binding, allowing formation of the ORP1-Rab7-RILP complex. ORP1 ANK binds to Rab7 and the Rab7-RILP complex with similar micro-molar affinities, which is consistent with the independence binding of ORP1 and RILP to Rab7. The structural model of the ORP1-Rab7-RILP complex correlates with the recruitment of ORP1 at the LEL-ER interface and the role in lipid transport and regulation.

## Introduction

The proper intracellular distribution of sterols and other lipids are crucial for the membrane identity and function. Lipoproteins taken up by endocytosis finally arrive at the lysosomes for degradation and release of free cholesterol [1]. Low-density lipoprotein (LDL)-derived cholesterol is redistributed to the plasma membrane by vesicular trafficking or to the ER by non-vesicular transport involving lipid transfer proteins (LTPs) [2,3]. Lipid transport mediated by the oxysterol-binding protein (OSBP)-related protein (ORPs) has been proposed as a major route of transporting of sterols and other phospholipids between intracellular membranes [4]. Many ORPs localize at the contact sites between the ER and other subcellular membranes and promote the exchange of specific lipids and regulate many biological processes [5]. Human has 12 ORP genes with four additional protein products by splicing variations [6]. The majority of ORPs contain multiple targeting domains such ankyrin (ANK), pleckstrin homology (PH), and a FFAT motif, in addition to the C-terminal lipid-binding OSBP-related domain (ORD) [7]. The ORD domains of all ORPs known to bind PI(4)P as a primary ligand and some ORPs also bind sterols or phosphatidylserine (PS) in a competitive manner [8–11]. In addition, several human ORPs including ORP2, ORP5, and ORP8 were known to bind and transport other phosphoinositides such as PI(4,5)P_2_ or PI(3,5)P_2_ [12–14]. Many ORPs transport sterols or PS against its concentration gradient between organellar membranes by using a PI(4)P gradient as an energy source [15,16].

ORP1L, a long ORP1 variant (hereafter referred to as ORP1), is specifically localized to the contact sites between the late endosomes/lysosomes (LELs) and the ER and implicated in non-vesicular sterol transport [3,17,18]. The N-terminal ANK domain targets ORP1 to the LELs via interaction with the Rab7 anchored to the endosomal membrane. [19]. Rab proteins which constitute a large family in the Ras-like GTPase superfamily, switch between active GTP-bound and inactive GDP-bound states and regulate diverse vesicular trafficking events [20]. Rab7 functions as a key regulator in maturation of endosomes and autophagosomes, directing the trafficking of cargos along microtubules, and in the fusion step with lysosomes, through different protein-protein interaction cascades [20,21]. The effector proteins of Rab7 such as Rab7-interacting lysosomal protein (RILP) and Rubicon associate only with the GTP-bound form of Rab7 [22,23]. ORP1 was known to regulate the mobility and distribution of late endosomes via connection with the RILP and dynein/dynactin motor complexes [19,24–26]. ORP1 was also suggested to function as a cholesterol transporter between the ER and the endo-lysosomal system [3,18].

Elucidating the structural mechanism of ORP1 recruitment to the LEL-ER interface is important for understating the inter-organelle communication and exchange at the contact sites. Here we characterized the structural and molecular details of the human ORP1 ANK–Rab7 interaction by determination of the crystal structure and biochemical analysis of the interaction. ORP1 ANK binds to the Rab7 surface which located away from the switch regions. This binding mode allows the association of RILP with the switch regions of the ORP1-bound Rab7. This study provides a framework for understanding the molecular basis of the ORP1–Rab7 interaction and how Rab7 GTPase recruits RILP and ORP1 simultaneously to the LEL membrane for lipid transport and regulation.

## Materials and methods

### 1. Cloning of human ORP1 ANK, Rab7, and RILP

The genes for human Rab7 (hMU001574), and RILP (hMU004337), were purchased from 21C Frontier Human Gene Bank (KRIBB, Daejeon, Republic of Korea). The DNA for ORP1 (UniProt ID: Q9BXW6) was amplified from human cDNA library. The DNA encoding the ORP1 ANK domain (residue 5–152 and residue 5–233) was sub-cloned into the BamHI/XhoI site of the modified pHIS-2 vector, in which the original TEV protease cleavage site was modified to a thrombin cleavage site [27]. The ORP1 ANK was tagged with an N-terminal hexa-histidine, followed by a thrombin protease cleavage site (LVPR/GS). The DNA encoding human Rab7 (residue 2–195) (UniProt ID: P51149) was sub-cloned into the BamHI/XhoI site of the modified pHIS-2 vector. The dominant active (Q67L) and dominant negative (T22N) mutants of Rab7 were prepared by point mutagenesis. All mutations were confirmed by DNA sequencing. The DNA encoding the coiled-coil region (residue 242–320) of human RILP (UniProt ID: Q96NA2) was cloned to BamHI/SalI site of the modified pHIS-2 vector.

### 2. Protein purification

*Escherichia coli* BL21(DE3) cells transformed with the plasmid encoding the ORP1 ANK, Rab7, or RILP were grown to an OD_600_ of 0.8 at 37°C. Cells were induced by the addition of isopropyl β-D-1-thiogalactopyranoside to a final concentration of 0.25 mM and were incubated for 12 h at 20°C before harvesting. Cells were resuspended in 2× PBS buffer (pH 7.5) containing 30 mM imidazole and lysed by sonication. After centrifugation, the supernatant containing the His-tagged recombinant protein was applied to a Ni-NTA affinity column. The protein was eluted from the column using 0.1 M Tris·HCl at pH 7.0, 0.3 M NaCl, 0.3 M imidazole. The eluted protein was concentrated to 10 mg/ml, and the His-tag was cleaved by addition of thrombin protease. The target protein was subjected to size exclusion chromatography (SEC) on a Superdex 200 column (GE healthcare) equilibrated with 20 mM Tris·HCl at pH 7.5, 150 mM NaCl. For the purification of the ORP1-Rab7 complex, the ORP1 ANK and Rab7 eluted from the Ni-NTA chromatography were mixed in a 1:1 molar ratio and the mixture was applied to SEC to isolate the ORP1-Rab7 complex. The fractions containing ORP1 ANK-Rab7 complex were concentrated to 10 mg/ml. In addition, 0.5 mM of MgCl_2_ and GTP were added to the purified protein prior to crystallization.

### 3. Crystallization and crystallographic analysis

Preliminary crystallization experiments for the ORP1 ANK-Rab7 complex were carried out in 96-well crystallization plates by hanging-drop vapor-diffusion method at 22°C. We used a truncated Rab7 construct (residue 2–195) with a dominant active mutation Q67L for the crystallization of the ORP1-Rab7 complex. However, we could not obtain crystals of the ORP1-Rab7 Q67L complex. To improve the crystallization properties of the ORP1-Rab7 complex by surface entropy reduction, we deleted the Leu73 residue (Δ73) in the flexible switch II region of Rab7. The Leu73 deletion was not necessary for the crystallization and structure determination of mouse ORP1 ANK -Rab7 [28]. The structural comparison of human and mouse structures showed that the effect of Δ73 is limited to the local conformation of switch II region (**S1A Fig**). The different requirement seems to originate from the different crystal lattice interactions. In the crystal of human ORP1-Rab7, the truncated switch II region (Δ73) composes a tight crystal lattice interaction, while Leu73 is more exposed to solvent channel in the crystal of mouse ORP1-Rab7 complex (**S1B Fig**). The Rab7 Q67L Δ73 mutant associated to ORP1 ANK similar to wild type suggesting that the mutation did not interfere with the protein-protein interaction. Crystals of the ORP1 ANK—Rab7 (Q67L, Δ73) complex with the dimensions of 0.15 × 0.15 × 0.1 mm appeared in 3 days in a crystallization solution containing 0.1 M Na_3_Citrate·HCl at pH 5.0, 1 M LiCl, 20% PEG 1000. The crystals were harvested and analyzed by SDS-PAGE to confirm the protein complex. The crystals were cryo-protected in Paratone oil, frozen by immersion in liquid nitrogen. Crystals were preserved in a cryogenic N_2_-gas stream (100 K) during diffraction experiments. Diffraction data for native crystals were collected to 2.1 Å resolution at a fixed wavelength of 0.97949 Å using an ADSC Q270 CCD detector on the 7A beamline at Pohang Light Source (PLS), Pohang Accelerator Laboratory, South Korea. All data were processed and scaled using HKL-2000. The phase was solved by molecular replacement using the structure of a GTP-bound Rab7 (PDB ID: 1T91) as a search model using software Phaser [29]. The ORP1 ANK was clearly visible in the 2*F*_o_-*F*_c_ electron density maps. Modeling was performed using the software COOT [30], and the structures were refined using Phenix [31]. The statistics for the X-ray crystallographic studies are shown in Table 1.

**Table 1:** Data-collection and refinement statistics.

Crystal	ORP1 –Rab7
Construct	Rab7 2–195, ORP1 5–152
Data collection	2016 0314 na12
Beamline	PLS-7A
Wavelength (Å)	0.97950
Space group	*P*2_1_2_1_2_1_
Unit-cell parameters (Å,°)	*a* = 55.1, *b* = 117.2, *c* = 130.5
Resolution range (Å)	50–2.1 (2.14–2.10)
No. of reflections	282965
No. of unique reflections	50042 (2496)
Multiplicity	5.7 (5.7)
Mean *I*/*σ*(*I*)	39.3 (7.3)
Completeness (%)	99.1 (99.9)
*R*_merge_ (%)	6.9 (32.4)
Wilson *B* factor (Å)	29.7
Refinement	
*R*_work_ (%)	19.5 (23.1)
*R*_free_ (%)	23.9 (27.2)
R.m.s.d., bond lengths (Å)	0.008
R.m.s.d., bond angles (°)	0.926
*B* factor (Å^2^)	
Overall	41.3
Molecule A, B (Rab7)	28.1, 57.3
Molecule C, D (ORP1)	45.1, 37.1
Ligands (GTP)	18.6, 43.1
Water	41.3
No. of non-H atoms	
Protein	4825
Ligand	66
Solvent	334
Ramachandran statistics	
Favored (%)	97.5
Disallowed (%)	0.0
PDB entry	6IYB

### 4. Isothermal titration calorimetry

To measure the affinity between ORP1 ANK and Rab7, isothermal titration calorimetry (ITC) was performed using an Affinity ITC calorimeter (low volume cell 190 μl; TA instruments) at 20°C. All proteins were prepared in the same buffer containing 20 mM Tris-HCl at pH 7.5, 150 mM NaCl. 100 μl of 1 mM ORP1 ANK protein was loaded into the syringe, and 177 μl of 0.1 mM Rab7 or Rab7-RILP was placed in the cell. Rab7 Q67L and Rab7 T22N were loaded with 0.5 mM of GTP and GDP, respectively. The titration curve was obtained by injecting 2 μl × 25 aliquots of ORP1 ANK into the cell for interaction, at a time interval of 200 sec. The enthalpy of reaction, ΔH^0^, the binding constant, K_d_, and the stoichiometry value, n, were calculated from the measured heat changes, δH_i_, upon the association of ORP1 ANK and Rab7. The titration data were analyzed using the program NanoAnalyze (TA instruments) and fitted into an independent binding model.

## Results

### Structure determination of the ORP1 ANK-Rab7 complex

**Fig 1:**
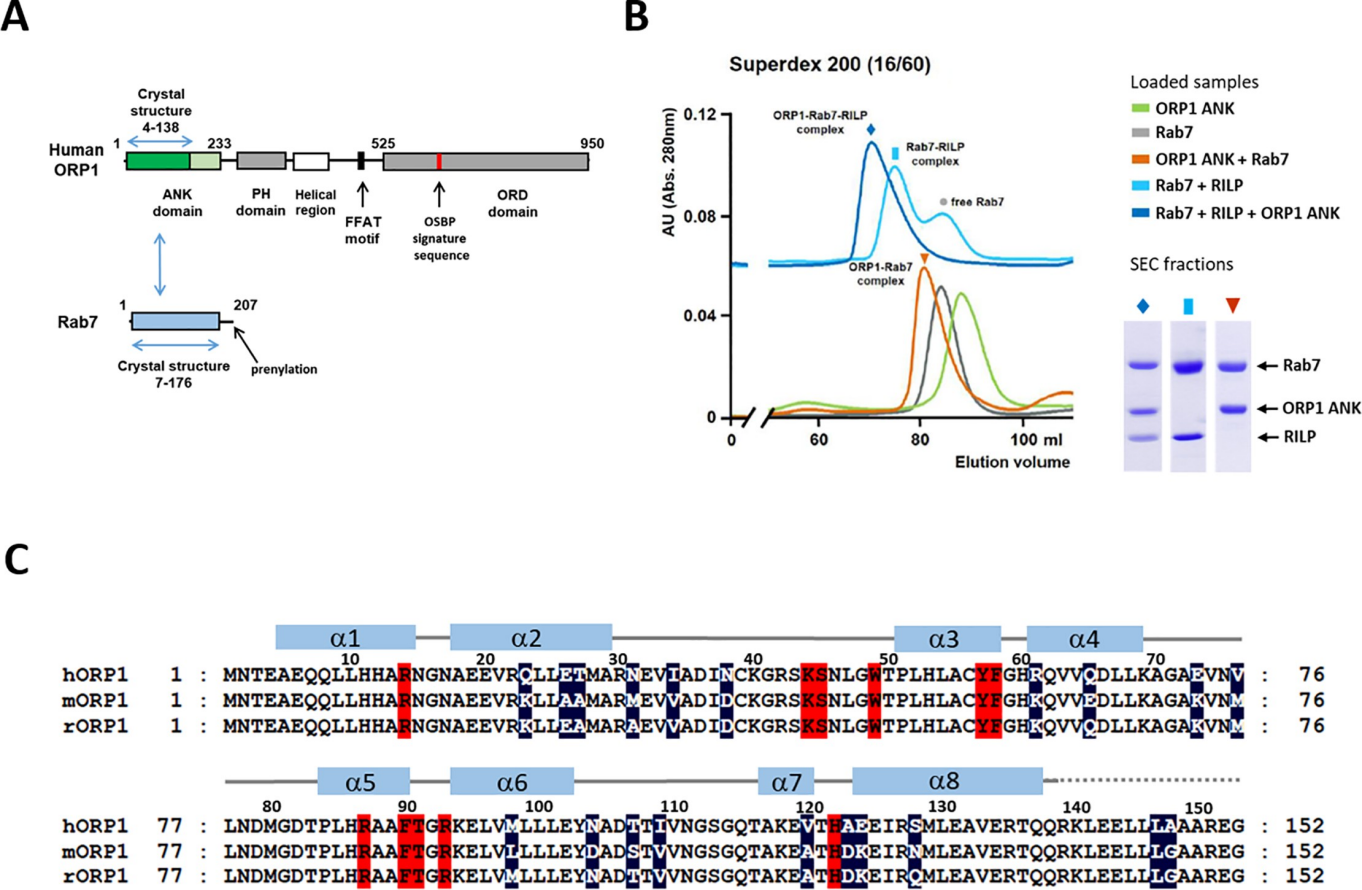
Association of ORP1 ANK and Rab7. (A) Schematic representation of the domain structures of human ORP1 and Rab7. The domains with the structures determined in this study are indicated with blue arrows. (B) Size exclusion chromatography (SEC) profiles of ORP1 ANK (5–152), Rab7 (2–195), RILP (241–320) and their mixtures. To analyze the protein-protein interaction, ORP1, Rab7, and RILP were mixed in a 1:1:1 molar ratio and incubated at room temperature for 30 min prior to SEC. Individual ORP1 ANK or Rab7 protein was applied separately as a control. The SDS-PAGE analysis of the major peaks are shown in the right panel. (C) Sequence alignments of the ANK domains from human, mouse, and rat ORP1 homologs. The residues forming the Rab7-binding sites are shaded in red. The variable residues in these species are shaded in dark blue.

Human ORP1 with 950 amino acids contains an N-terminal ANK domain, PH domain, FFAT motif and the C-terminal ORD domain which are connected by flexible loops (**Fig 1A**). The secondary structure prediction (https://www.predictprotein.org) suggested that human ORP1 has four or five ankyrin repeats in the N-terminal region. We purified human ORP1 ANK, Rab7, and RILP separately. To confirm the binding of ORP1 ANK to Rab7, we mixed with the purified ORP1 ANK and Rab7 and analyzed the complex formation by size exclusion chromatography (SEC) (**Fig 1B**). We observed that the small ORP1 ANK construct (residue 5–152) containing four ankyrin repeats is sufficient to associate with Rab7 (**Fig 1C**). GTP-loaded Rab7 associated with ORP1 ANK and RILP, and also formed a tripartite complex of ORP1-Rab7-RILP.

**Fig 2:**
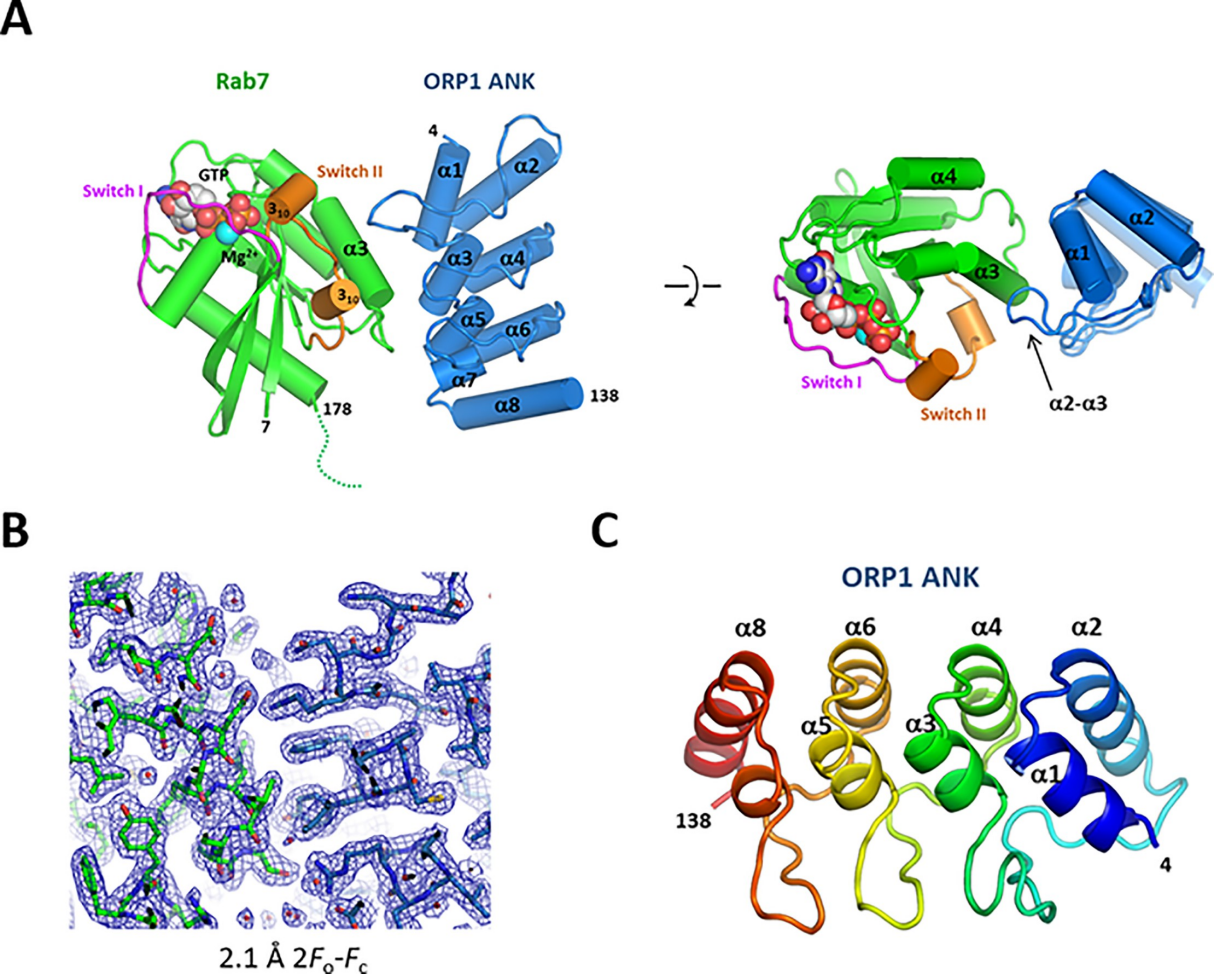
Overall structure of human ORP1 ANK and Rab7 complex. (A) Cylindrical representation of the ORP1-Rab7 complex. The bound GTP and Mg^2+^ ion are shown in spheres. The disordered C-terminus of Rab7 is indicated with a dotted line. Top view of the structure is shown in the right panel. (B) 2.1 Å 2*F*_0_-*F*c electron density maps with the final model superimposed. The Rab7 structure is shown in green and ORP1 ANK in blue. (C) Overall structure of human ORP1 ANK domain.

For crystallographic studies of the ORP1 ANK-Rab7 complex, the purified ORP1 ANK and Rab7 proteins were mixed in a 1:1 molar ratio, and subsequently, the ORP1-Rab7 complex was isolated by SEC. To inhibit GTP hydrolysis and to improve crystallization properties of the ORP1-Rab7 complex, we used a dominant active Q67L mutant of Rab7 and introduced a deletion of Leu73 (Δ73) at the start of the switch II region, which is the most disordered region in the GTP-bound form of Rab7 structures (PDB ids: 3LAW, 1T91) [23,32]. The Rab7 Q67L Δ73 mutant associated with ORP1 ANK suggesting that the switch II region is not involved in ORP1 binding. The structure of the ORP1 ANK–Rab7 complex was solved by molecular replacement using a structure of GTP-bound Rab7 (PDB id: 1T91). The bound GTP and Mg^2+^ ion in Rab7 were clearly visible in the 2.1 Å electron density maps. The N-terminal 6 and the C-terminal 17 residues of Rab7 (residue 179–195) were disordered and not visible. The electron densities of ORP1 ANK was well visible for model building (**Fig 2A and 2B**). The asymmetric unit of the crystal contained two heterodimers of the ORP1–Rab7 complex. The two heterodimers had almost identical structures with the C_α_ rmsd of 0.37 Å.

The human ORP1 ANK is composed of four helix-turn-helix repeats connected by a long or tight β-hairpin loop (**Fig 2C**). The ORP1 ANK shows a canonical structure of ankyrin domains with the L-shaped consecutive repeats stacked together to form an extended domain. The ANK repeat commonly contains a conserved TPLH tetrapeptide motif at the start of the inner α helix, which stabilizes the repeats by hydrogen bonding between threonine and histidine residues. ORP1 ANK contains two TPLH motifs in the helices α3 and α5 of the second and third ANK repeats, respectively. The β-hairpin loop of the first ANK repeat is extended with four additional residues compared to the second and third β-hairpin loops.

Rab7 has a typical GTPase fold which consists of a six-stranded β-sheet flanked by five α-helices. In the crystal structure, the GTP-bound Rab7 has a closed conformation of switch regions (**Fig 2A**). The structure of the GTP-bound Rab7 Q67L Δ73 was almost identical to the structure of Rab7-GTP Q67L (PDB id: 1T91) with the C_α_ rmsd of 0.30 Å except a small conformational difference originating from deletion of Leu73 in the switch II region.

### ORP1 ANK–Rab7 interaction

**Fig 3:**
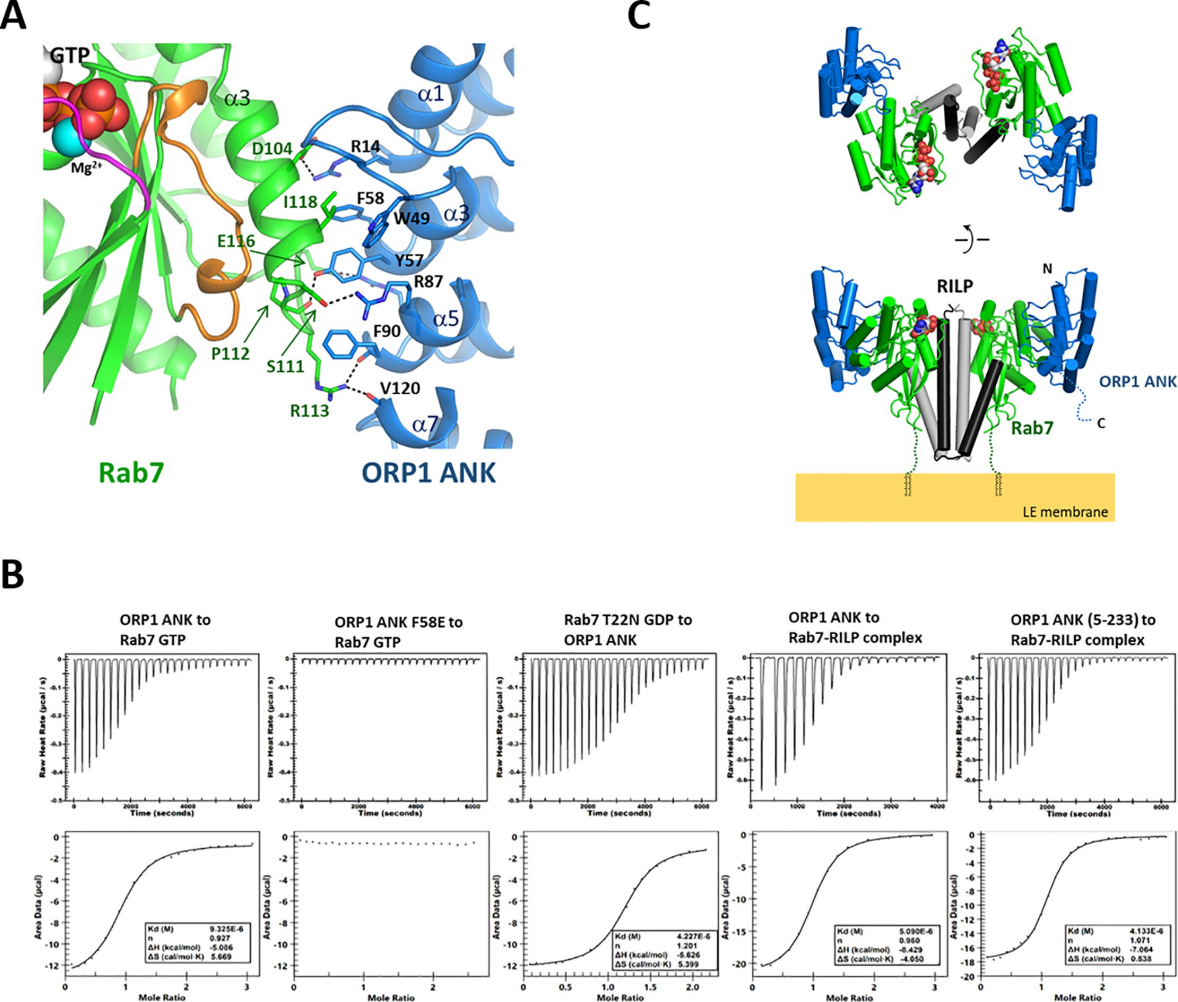
Formation of the ORP1-Rab7-RILP complex. (A) Binding interface of the ORP1 ANK and Rab7. The residues forming the interface are shown in stick models. The hydrogen bonds are shown in dashed lines. (B) Measurement of binding affinity of ORP1 ANK to Rab7 by isothermal titration calorimetry. The purified Rab7 or Rab7-RILP complex with the concentration of 0.1 mM was titrated with 1 mM of ORP1 ANK. Rab7 was either loaded with GTP or GDP. (C) Overall structure of the ORP1 ANK-Rab7-RILP complex. The structure was constructed by combining the structures of Rab7-RILP (PDB id: 1YHN) and ORP1 -Rab7 (this study) using PyMOL (https://pymol.org). The missing C-terminal residues (186–203) of Rab7 were indicated with dotted lines. The C-terminal prenyl groups which anchor Rab7 onto the endosomal membrane are shown in black lines.

ORP1 ANK binds to Rab7 using the concave surface composed of the inner helices of ANK repeats and the first β-hairpin loop. The helix α3 and the α3-β5 loop of Rab7 compose the ANK binding site. The nucleotide-binding site of Rab7 is located away from the ORP1-Rab7 interface. The switch I and switch II regions which undergo conformational changes upon GTP or GDP binding are away from the interface, indicating the independence of ORP1–Rab7 interaction from the nucleotide binding. The switch II region is 12 Å apart from the ORP1-Rab7 interface, and does not make a contact with ORP1 ANK. The binding interface is formed by 12 and 13 residues from ORP1 and Rab7, respectively. The interface area is 677 Å^2^ burying 8.4% of the total surface of the ORP1-Rab7 complex. The polar interaction composes 52% of the binding interface. Several basic residues such as Arg14, Arg87, and Arg93 of ORP1 make hydrogen bonds with Asp104, Ser111, and Glu116 of Rab7 (**Fig 3A**). The bulky hydrophobic residues including Trp49, Phe50, Tyr57, and Phe58 from ORP1 make hydrophobic interaction with the residues on the helix α3 and the α3-β5 loop of Rab7. The ORP1 ANK domains of human and mouse share 87% sequence identity with strict conservation of the ORP1-Rab7 interface (**Fig 1C**). Our observation is consistent with the structure of mouse ORP1 ANK-Rab7 complex (PDB id: 5Z2M) recently reported (**S1A Fig**) [28].

### ORP1 ANK–Rab7 interaction is independent of GTP- or GDP-binding state of Rab7

To analyze the interaction of ORP1 ANK and Rab7, we measured the affinity and stoichiometry of the protein binding by isothermal titration calorimetry (**Fig 3B**). ORP1 ANK bound to GTP-loaded Rab7 with a Kd of 9.3 μM with 1:1 stoichiometry. To confirm the ORP1-Rab7 interaction observed in the crystal structure, we mutated the residue Phe58 located in the center of the interface to Glu, and tested the binding of ORP1 F58E to Rab7. The F58E mutation completely abolished the interaction confirming the ORP1-Rab7 interaction observed in the crystal structure is consistent in solution. The Rab7 effector RILP binds only to the GTP-loaded Rab7 by recognizing the switch regions. To check the nucleotide dependency of the ORP1-Rab7 interaction, we checked the binding affinity of ORP1 ANK to the GDP-loaded form of Rab7 (T22N). The Rab7 T22N is a constitutive GDP-binding inactive mutant [33]. The ORP1 ANK bound GTP- or GDP-loaded forms of Rab7 with similar micro-molar affinities (**Fig 3B**). In addition, ORP1 ANK associated with the Rab7-RILP complex with a Kd of 5 μM, which is similar to the affinity of ORP1-Rab7 interaction. The ITC data are consistent with the crystal structure and SEC analysis, suggesting that the interaction of ORP1 ANK and Rab7 is independent of the nucleotide-binding state of Rab7, which allows formation of the ORP1-Rab7-RILP tripartite complex.

### Structure modeling of the ORP1-Rab7-RILP complex

ORP1L homolog is specifically targeted to the LEL membrane by its ANK domain [19]. Rab7 is anchored to the LEL membrane via prenylation of the two cysteine residues in the C-terminus. The Rab7-binding domain of RILP forms a tight coiled-coil homodimer, allowing symmetric binding of two separate Rab7 molecules simultaneously [23]. The structure of the ORP1-Rab7-RILP complex was modeled by combining the structures of Rab7-RILP (PDB id: 1YHN) and ORP1 ANK–Rab7 (This study). In this complex, a homodimer of RILP in the center interacts with two molecules of Rab7 in two-fold symmetry. ORP1 ANK associates with each Rab7 in 90 degree away from RILP and does not contact with RILP (**Fig 3C**). Therefore, ORP1 ANK binding is independent of the nucleotide-binding state of Rab7 and RILP binding. The C-termini of two Rab7 molecules in the complex are anchored to the LEL membrane, positioning the coiled-coil domain of RILP vertical to the membrane surface and ORP1 ANK away from the membrane, which demonstrates the spatial arrangement of the subunits required for membrane recruitment.

## Discussion

**Fig 4:**
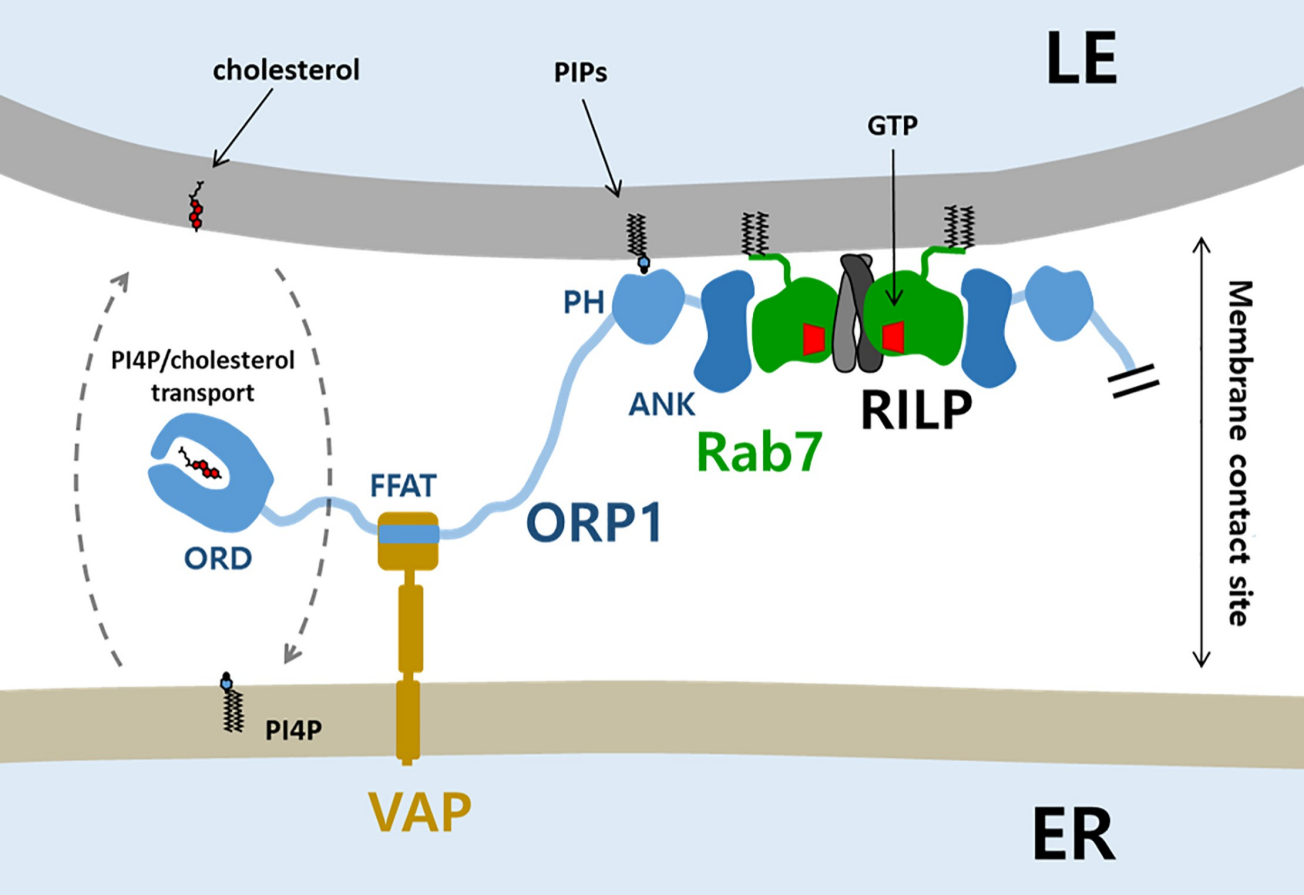
Schematic model of ORP1 function at the LEL-ER membrane contact site. The prenylated Rab7 localizes to the late-endosomal/lysosomal membrane. GTP-loaded Rab7 associated with the dimeric RILP recruits two molecules of ORP1 by ORP1 ANK-Rab7 interaction. The ORP1 has additional targeting modules such as a PH domain and a FFAT motif which target to the LEL-ER membrane contact sites. The ORD domain of ORP1 might transport PI4P and cholesterol between the membranes.

The endocytic pathway sorts and delivers low-density lipoproteins to LELs for the hydrolysis of cholesterol esters. The released cholesterol is moved to the limiting membrane of LELs by the actions of Niemann Pick C 1 and 2 (NPC1 and 2) proteins [34,35]. ORP1 was suggested to function as a lipid transporter that moves out cholesterol from the endo-lysosomal system to other organelles [3]. In contrast, when LDL-cholesterol in the endosomes is low, ORP1 promotes cholesterol transport from the ER to multivesicular bodies (MVBs) to support intraluminal vesicle formation [18]. Therefore, the direction of cholesterol transport by ORP1 might depend on the cholesterol concentration of the LEL and the ER. The cholesterol transport by ORP1 seems to be partly governed by the recruitment of ORP1 to the LEL-ER interface. ORP1L is basically a cytosolic protein. However, the majority of ORP1 is found on the LEL membranes [17]. The LEL localization of ORP1 is dictated by the association of ANK domain to the membrane-anchored Rab7. The prenylated Rab GTPases are present either on membranes or in the cytosol. The cytosolic Rab7 in an inactive form is bound tightly to a Rab-escort protein (REP) or GDP-dissociation inhibitor (GDI) for the REP/GDI-mediated delivery of prenylated Rab7 to membranes [36]. The REP and GDI binding sites in Rab7 partially overlap with the binding site of ORP1 ANK, preventing association of ORP1 to the cytosolic Rab7 (**S2 Fig**). Therefore, only the Rab7 present in the LEL membranes recruits ORP1. The PH domain enhances the membrane association of ORP1 and the ER-targeting FFAT motif allows the localization of ORP1 to the LEL-ER contact site [3,17]. Due to the dual membrane targeting of ORP1, lipid transport function of ORP1 seems to be effective at the close membrane contact sites with a typical separation of less than 10–30 nm [37] (**Fig 4**).

Of human ORPs, only ORP1L homolog has an ANK domain. In yeast Osh homologs, Osh1 and Osh2 contain the N-terminal ANK domains. The yeast Osh1 ANK displays a bi-lobed structure with two ANK domains connected by a central α helix [38]. The Osh1 ANK domain targets Osh1 to the nucleus-vacuole junctions by association with the nuclear membrane protein Nvj1 [39]. There is no structural and functional correlation between the ANK domains of human and yeast ORPs. However, the both ANK domains mediate protein-targeting to specific subcellular membranes by their unique protein-protein interactions. ORP1 was initially known to interact preferentially with the GTP-bound form Rab7 and regulate the juxta nuclear clustering of late endosomes [19]. However, the recent structural and biochemical studies of mouse ORP1-Rab7 interaction suggested that the interaction is independent of the nucleotide-binding states of Rab7 [28]. The structure of human ORP1-Rab7 in this study is fully consistent with the findings by Ma et al [28]. ORP1 ANK binds to the non-canonical site of Rab7 away from the switch regions, suggesting that the ORP1-Rab7 interaction is independent of the binding of Rab7 effector proteins, implying that the major role of the protein-protein interaction is recruitment of ORP1 to the specific site.

Phosphoinositides and cholesterol in the limiting membranes of LELs are important for the regulation of the movement and function of LELs [40,41]. Considering the general function of ORPs in lipid transport, ORP1 recruited by Rab7 might transport cholesterol required for the late endosome maturation and processing. Many ORPs were known to transport sterol or PS against their concentration gradients using a phosphoinositide gradient as an energy source, transporting cholesterol in the ER to other membranes. However, the mechanism of lipid transport by ORP1 at the LEL-ER interface has remained elusive and requires further investigation. In conclusion, this study demonstrates the key determinants of ORP1 recruitment through Rab7 interaction at the membrane contact sites, which is required for the lipid transport and regulatory role of ORP1.

## Supporting information

S1 Fig Structure comparison of human and mouse ORP1-Rab7 complexes. (A) The structures of human and mouse ORP1-Rab7 complexes were superimposed. The switch II region of human Rab7 has a slightly different conformation compared to the structure of mouse ORP1-Rab7 due to Δ73 mutation. (B) Crystal lattice interactions of human and mouse ORP1-Rab7 structures. In the crystal of human ORP1-Rab7, the truncated switch II region with (Δ73) composes the tight crystal lattice interaction, indicating that the surface entropy reduction by L73Δ was critical for crystallization. In contrast, Δ73 was not necessary for the crystallization of mouse ORP1-Rab7 due to the difference lattice interaction.
(TIF)

S2 Fig ORP1 does not bind the cytosolic Rab7. The structures of human ORP1 ANK (surface representation) were positioned to the structures of REP-1-Rab7 (PDB id: 1VG9) and GDI-Ypt1 (PDB id: 2BCG). The cytosolic Rab7 in an inactive form is bound tightly to a GDP-dissociation inhibitor (GDI) and Rab-escort protein (REP). The GDI and REP binding sites in Rab7 partially overlap with the binding site of ORP1 ANK, preventing association of ORP1 to the cytosolic Rab7. Therefore, only the Rab7 present in the LEL membranes recruits ORP1.
(TIF)

S1 File Uncropped SDS-PAGE gel images for Fig 1B. (DOCX)

S2 File PDB X-ray structure validation report. (PDF)
