# Supplementary figures and images for: Structural basis of human ORP1-Rab7 interaction for the late-endosome and lysosome targeting
Source: PLoS One. 2019 Feb 5;14(2):e0211724. doi: 10.1371/journal.pone.0211724 (PMC6363164; doi:10.1371/journal.pone.0211724)

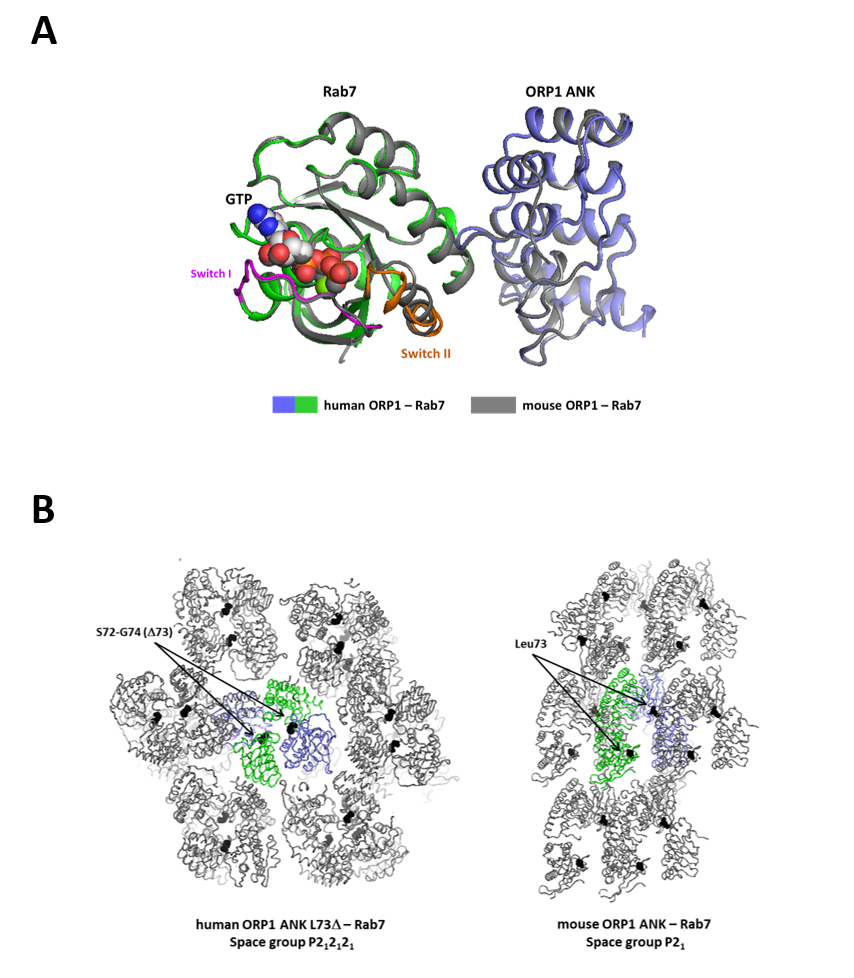

Supplement: S1 Fig — (A) The structures of human and mouse ORP1-Rab7 complexes were superimposed. The switch II region of human Rab7 has a slightly different conformation compared to the structure of mouse ORP1-Rab7 due to Δ73 mutation. (B) Crystal lattice interactions of human and mouse ORP1-Rab7 structures. In the crystal of human ORP1-Rab7, the truncated switch II region with (Δ73) composes the tight crystal lattice interaction, indicating that the surface entropy reduction by L73Δ was critical for crystallization. In contrast, Δ73 was not necessary for the crystallization of mouse ORP1-Rab7 due to the difference lattice interaction. (TIF) [file pone.0211724.s001.tif]

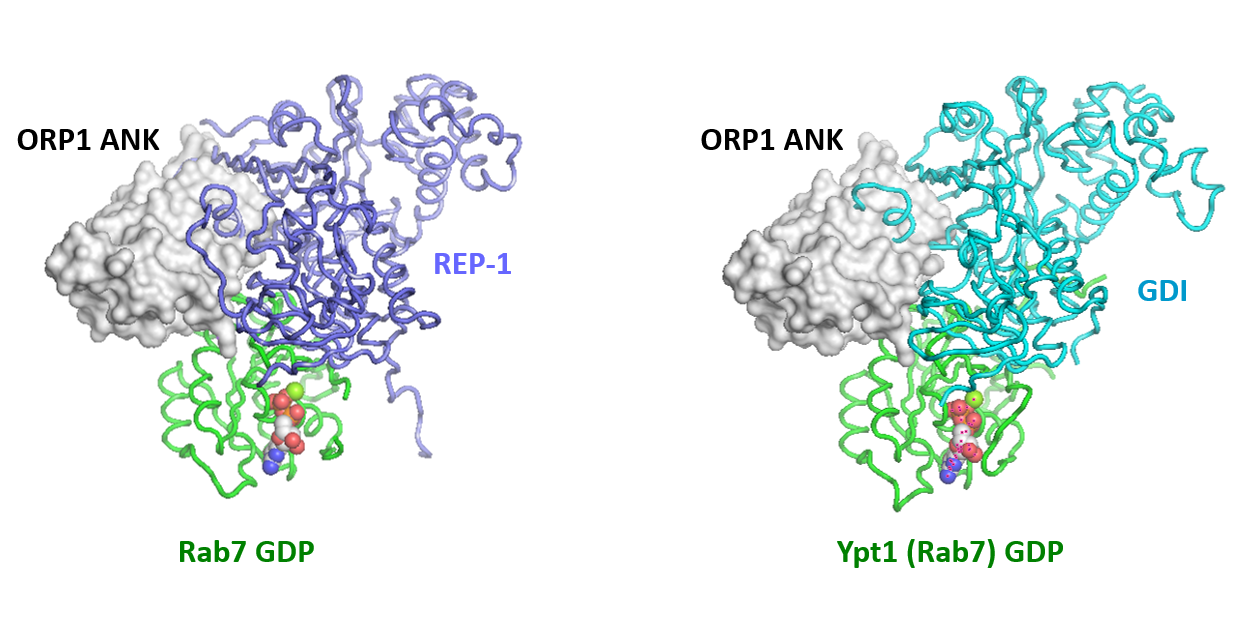

Supplement: S2 Fig — The structures of human ORP1 ANK (surface representation) were positioned to the structures of REP-1-Rab7 (PDB id: 1VG9) and GDI-Ypt1 (PDB id: 2BCG). The cytosolic Rab7 in an inactive form is bound tightly to a GDP-dissociation inhibitor (GDI) and Rab-escort protein (REP). The GDI and REP binding sites in Rab7 partially overlap with the binding site of ORP1 ANK, preventing association of ORP1 to the cytosolic Rab7. Therefore, only the Rab7 present in the LEL membranes recruits ORP1. (TIF) [file pone.0211724.s002.tif]
