# Supplementary material for: Structural basis of human ORP1-Rab7 interaction for the late-endosome and lysosome targeting
Source: PLoS One. 2019 Feb 5;14(2):e0211724. doi: 10.1371/journal.pone.0211724 (PMC6363164; doi:10.1371/journal.pone.0211724)

**Supporting information**

Uncropped SDS-PAGE gel images for figure 1B

ORP1 ANK-Rab7-RILP complex


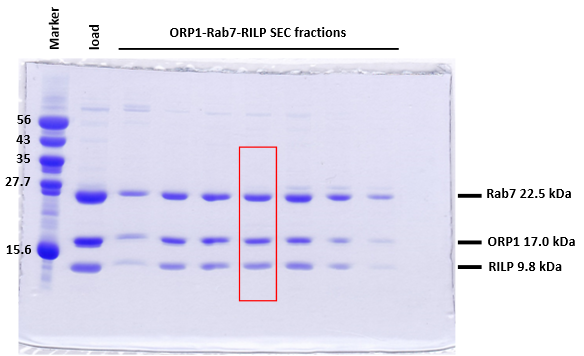


Rab7-RILP complex


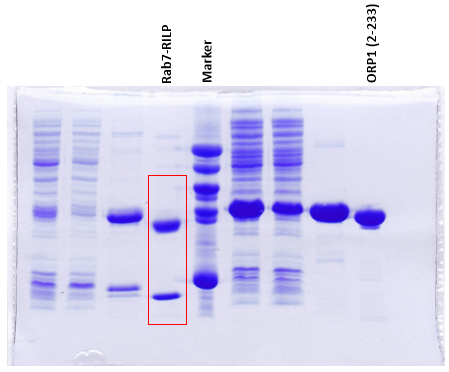


ORP1 ANK-Rab7 complex


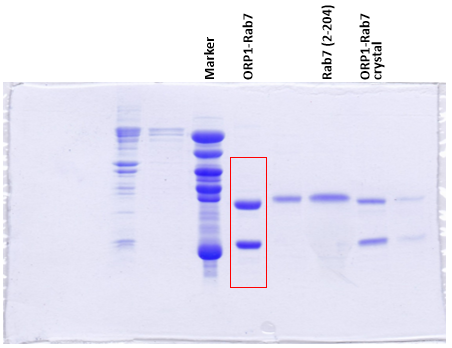

Supplement: S1 File — (DOCX) [file pone.0211724.s003.docx]
