# Supplementary material for: Structural basis of human ORP1-Rab7 interaction for the late-endosome and lysosome targeting
Source: PLoS One. 2019 Feb 5;14(2):e0211724. doi: 10.1371/journal.pone.0211724 (PMC6363164; doi:10.1371/journal.pone.0211724)
Supplement: S2 File — (PDF) [file pone.0211724.s004.pdf]

Preliminary Full wwPDB X-ray Structure Validation Report (i)

Dec 14, 2018 – 04:22 PM JST

Deposition ID	:	D_1300010156
PDB ID	:	*(not yet assigned)*

This is a Preliminary Full wwPDB X-ray Structure Validation Report.

This report is produced by the wwPDB Deposition System during initial deposition but before annotation of the structure.

We welcome your comments at *validation@mail.wwpdb.org*
A user guide is available at
https://www.wwpdb.org/validation/2017/XrayValidationReportHelp
with specific help available everywhere you see the (i) symbol.

PRELIMINARY VALIDATION REPORT

The following versions of software and data (see references (i)) were used in the production of this report:

MolProbity	:	4.02b-467
Mogul	:	1.7.3 (157068), CSD as539be (2018)
Xtriage (Phenix)	:	1.13
EDS	:	rb-20031633
Percentile statistics	:	20171227.v01 (using entries in the PDB archive December 27th 2017)
Refmac	:	5.8.0158
CCP4	:	7.0 (Gargrove)
Ideal geometry (proteins)	:	Engh & Huber (2001)
Ideal geometry (DNA, RNA)	:	Parkinson et al. (1996)
Validation Pipeline (wwPDB-VP)	:	rb-20031633

1 Overall quality at a glance (i)

The following experimental techniques were used to determine the structure:
X-RAY DIFFRACTION

The reported resolution of this entry is 2.09 Å.

Percentile scores (ranging between 0-100) for global validation metrics of the entry are shown in the following graphic. The table shows the number of entries on which the scores are based.

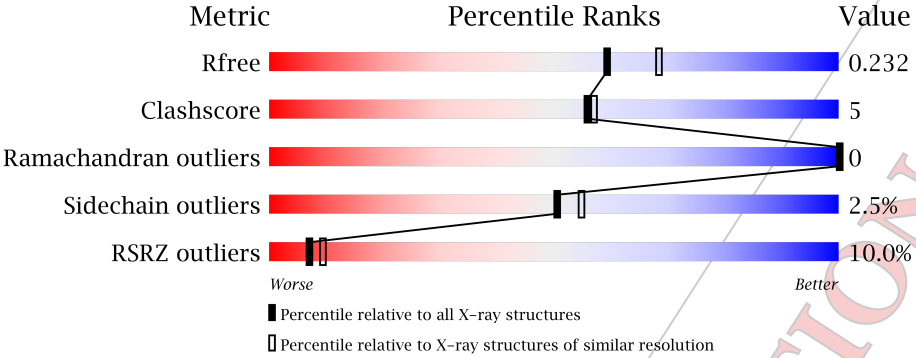

Metric	Whole archive (#Entries)	Similar resolution (#Entries, resolution range(Å))
R_{free}	111664	4608 (2.10-2.10)
Clashscore	122126	5109 (2.10-2.10)
Ramachandran outliers	120053	5059 (2.10-2.10)
Sidechain outliers	120020	5060 (2.10-2.10)
RSRZ outliers	108989	4497 (2.10-2.10)

The table below summarises the geometric issues observed across the polymeric chains and their fit to the electron density. The red, orange, yellow and green segments on the lower bar indicate the fraction of residues that contain outliers for >=3, 2, 1 and 0 types of geometric quality criteria. A grey segment represents the fraction of residues that are not modelled. The numeric value for each fraction is indicated below the corresponding segment, with a dot representing fractions <=5% The upper red bar (where present) indicates the fraction of residues that have poor fit to the electron density. The numeric value is given above the bar.

Mol	Chain	Length	Quality of chain
1	A	199	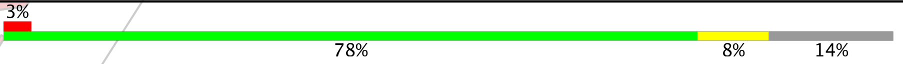
1	C	199	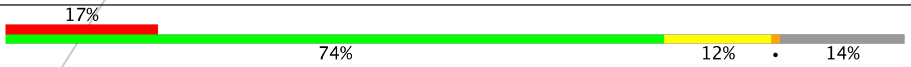
2	B	135	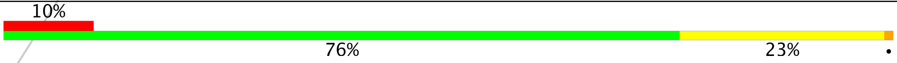
3	D	135	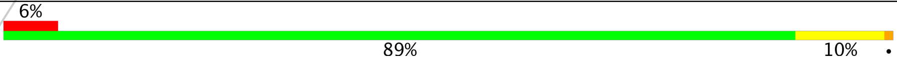

PRELIMINARY VALIDATION REPORT

2 Entry composition (i)

There are 6 unique types of molecules in this entry. The entry contains 5225 atoms, of which 0 are hydrogens and 0 are deuteriums.

In the tables below, the ZeroOcc column contains the number of atoms modelled with zero occupancy, the AltConf column contains the number of residues with at least one atom in alternate conformation and the Trace column contains the number of residues modelled with at most 2 atoms.

- Molecule 1 is a protein called Rab7.

Mol	Chain	Residues	Atoms	ZeroOcc	AltConf	Trace
1	A	171	Total C N O S<br>1363 869 231 257 6	0	0	0
1	C	171	Total C N O S<br>1363 869 231 257 6	0	0	0

- Molecule 2 is a protein.

Mol	Chain	Residues	Atoms	ZeroOcc	AltConf	Trace
2	B	135	Total C N O S<br>1052 645 197 204 6	0	0	0

- Molecule 3 is a protein.

Mol	Chain	Residues	Atoms	ZeroOcc	AltConf	Trace
3	D	135	Total C N O S<br>1047 642 196 203 6	0	0	0

- Molecule 4 is MAGNESIUM ION (three-letter code: MG) (formula: Mg).

Mol	Chain	Residues	Atoms	ZeroOcc	AltConf
4	E	1	Total Mg<br>1 1	0	0
4	F	1	Total Mg<br>1 1	0	0

- Molecule 5 is GUANOSINE-5'-TRIPHOSPHATE (three-letter code: GTP) (formula: $C_{10}H_{16}N_5O_{14}P_3$).

PRELIMINARY VALIDATION REPORT

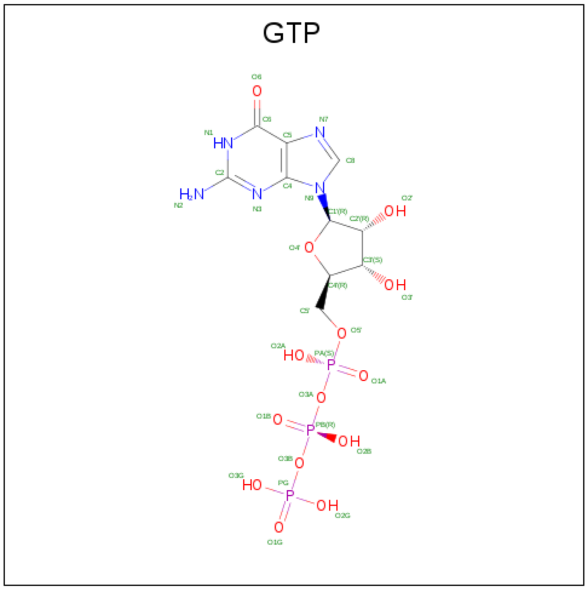

Mol	Chain	Residues	Atoms	ZeroOcc	AltConf
5	E	1	Total C N O P<br>32 10 5 14 3	0	0
5	F	1	Total C N O P<br>32 10 5 14 3	0	0

- Molecule 6 is water.

Mol	Chain	Residues	Atoms	ZeroOcc	AltConf
6	S	334	Total O<br>334 334	0	0

PRELIMINARY VALIDATION REPORT

3 Residue-property plots (i)

These plots are drawn for all protein, RNA and DNA chains in the entry. The first graphic for a chain summarises the proportions of the various outlier classes displayed in the second graphic. The second graphic shows the sequence view annotated by issues in geometryand electron density. Residues are color-coded according to the number of geometric quality criteria for which they contain at least one outlier: green = 0, yellow = 1, orange = 2 and red = 3 or more. A red dot above a residue indicates a poor fit to the electron density (RSRZ > 2). Stretches of 2 or more consecutive residues without any outlier are shown as a green connector. Residues present in the sample, but not in the model, are shown in grey.

- Molecule 1: Rab7

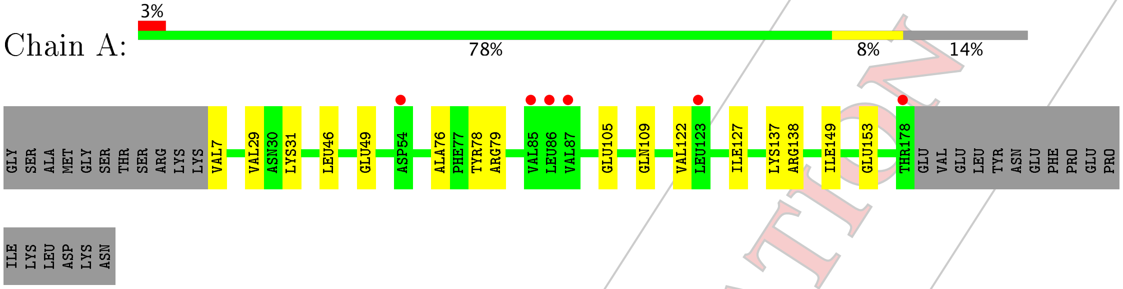

- Molecule 1: Rab7

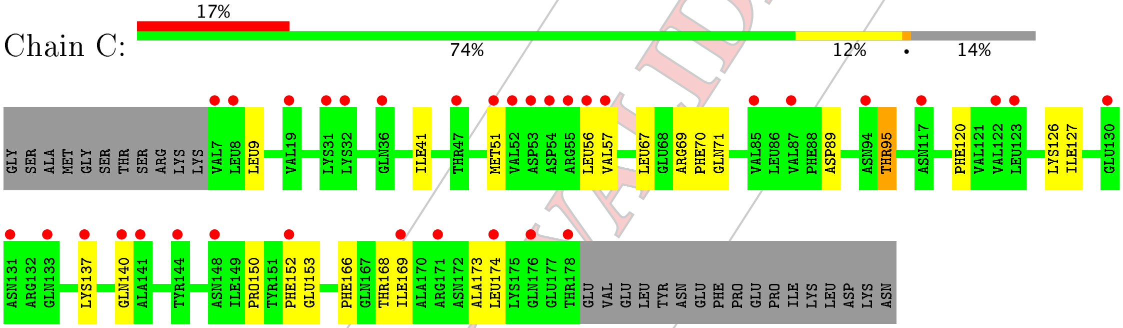

- Molecule 2:

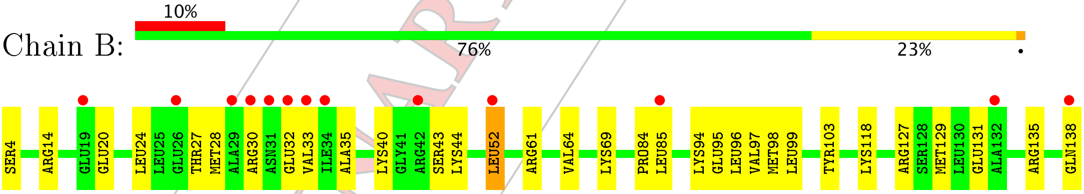

- Molecule 3:

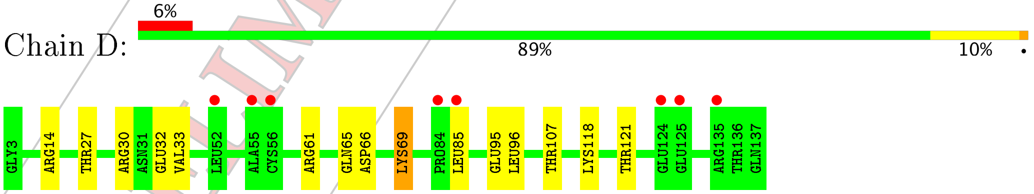

PRELIMINARY VALIDATION REPORT

4 Data and refinement statistics (i)

Property	Value	Source
Space group	P 21 21 21	Depositor
Cell constants<br>a, b, c, α, β, γ	55.14Å 117.22Å 130.48Å<br>90.00° 90.00° 90.00°	Depositor
Resolution (Å)	26.29 – 2.09<br>26.29 – 2.09	Depositor<br>EDS
% Data completeness<br>(in resolution range)	98.5 (26.29-2.09)<br>95.5 (26.29-2.09)	Depositor<br>EDS
R_{merge}	(Not available)	Depositor
R_{sym}	(Not available)	Depositor
$< I/\sigma(I) >$ [1]	6.38 (at 2.10Å)	Xtriage
Refinement program	PHENIX (1.10.1_2155: ???)	Depositor
R, R_{free}	0.195 , 0.232<br>0.195 , 0.232	Depositor<br>DCC
R_{free} test set	2545 reflections (5.09%)	wwPDB-VP
Wilson B-factor (Å^2)	29.0	Xtriage
Anisotropy	0.278	Xtriage
Bulk solvent k_{sol}(e/Å^3), B_{sol}(Å^2)	0.39 , 56.9	EDS
L-test for twinning[2]	$< \|L\| >$ = 0.49, $< L^2 >$ = 0.32	Xtriage
Estimated twinning fraction	No twinning to report.	Xtriage
F_o,F_c correlation	0.94	EDS
Total number of atoms	5225	wwPDB-VP
Average B, all atoms (Å^2)	41.0	wwPDB-VP

Xtriage's analysis on translational NCS is as follows: *The largest off-origin peak in the Patterson function is 4.08% of the height of the origin peak. No significant pseudotranslation is detected.*

[1] Intensities estimated from amplitudes.

[2] Theoretical values of $< |L| >$, $< L^2 >$ for acentric reflections are 0.5, 0.333 respectively for untwinned datasets, and 0.375, 0.2 for perfectly twinned datasets.

PRELIMINARY VALIDATION REPORT

5 Model quality (i)

5.1 Standard geometry (i)

Bond lengths and bond angles in the following residue types are not validated in this section: GTP, MG

The Z score for a bond length (or angle) is the number of standard deviations the observed value is removed from the expected value. A bond length (or angle) with $|Z| > 5$ is considered an outlier worth inspection. RMSZ is the root-mean-square of all Z scores of the bond lengths (or angles).

Mol	Chain	Bond lengths		Bond angles	
		RMSZ	$\#\|Z\| >5$	RMSZ	$\#\|Z\| >5$
1	A	0.52	0/1389	0.62	0/1881
1	C	0.35	0/1389	0.50	0/1881
2	B	0.43	0/1065	0.57	0/1438
3	D	0.43	0/1060	0.54	0/1431
All	All	0.44	0/4903	0.56	0/6631

There are no bond length outliers.

There are no bond angle outliers.

There are no chirality outliers.

There are no planarity outliers.

5.2 Too-close contacts (i)

In the following table, the Non-H and H(model) columns list the number of non-hydrogen atoms and hydrogen atoms in the chain respectively. The H(added) column lists the number of hydrogen atoms added and optimized by MolProbity. The Clashes column lists the number of clashes within the asymmetric unit, whereas Symm-Clashes lists symmetry related clashes.

Mol	Chain	Non-H	H(model)	H(added)	Clashes	Symm-Clashes
1	A	1363	0	1352	12	1
1	C	1363	0	1352	13	0
2	B	1052	0	1040	23	0
3	D	1047	0	1035	8	0
4	E	1	0	0	0	0
4	F	1	0	0	0	0
5	E	32	0	12	0	0
5	F	32	0	12	1	0
6	S	334	0	0	7	1
All	All	5225	0	4803	53	1

PRELIMINARY VALIDATION REPORT

The all-atom clashscore is defined as the number of clashes found per 1000 atoms (including hydrogen atoms). The all-atom clashscore for this structure is 5.

All (53) close contacts within the same asymmetric unit are listed below, sorted by their clash magnitude.

Atom-1	Atom-2	Interatomic distance (Å)	Clash overlap (Å)
2:B:94:LYS:NZ	6:S:226:HOH:O	2.12	0.79
2:B:4:SER:N	6:S:257:HOH:O	2.16	0.77
2:B:30:ARG:NH1	2:B:32:GLU:OE2	2.18	0.76
1:A:78:TYR:OH	6:S:319:HOH:O	2.03	0.76
1:A:29:VAL:HG21	1:A:46:LEU:HD11	1.71	0.72
3:D:61:ARG:NH1	3:D:65:GLN:OE1	2.25	0.69
1:A:105:GLU:OE2	6:S:319:HOH:O	2.10	0.69
2:B:61:ARG:HA	2:B:96:LEU:HD21	1.74	0.69
2:B:30:ARG:O	2:B:30:ARG:HG2	1.93	0.68
1:A:105:GLU:HB2	2:B:44:LYS:HG3	1.76	0.67
2:B:28:MET:HG2	2:B:35:ALA:HB3	1.77	0.65
2:B:131:GLU:OE2	2:B:135:ARG:NH2	2.30	0.63
2:B:52:LEU:HD13	2:B:84:PRO:HG2	1.78	0.63
2:B:61:ARG:HD3	2:B:95:GLU:OE1	2.01	0.60
1:A:105:GLU:OE2	2:B:44:LYS:HD2	2.01	0.60
1:C:127:ILE:HG21	1:C:153:GLU:HB3	1.84	0.58
3:D:27:THR:HG22	3:D:33:VAL:HG22	1.86	0.56
1:A:109:GLN:NE2	6:S:175:HOH:O	2.39	0.56
3:D:61:ARG:HH12	3:D:65:GLN:CD	2.11	0.54
1:A:127:ILE:HG21	1:A:153:GLU:HB3	1.90	0.53
2:B:27:THR:HG22	2:B:33:VAL:HG22	1.91	0.53
2:B:98:MET:CE	2:B:129:MET:HG2	2.40	0.52
3:D:61:ARG:HA	3:D:96:LEU:HD21	1.94	0.49
3:D:66:ASP:O	3:D:69:LYS:HE3	2.12	0.49
2:B:94:LYS:H	2:B:94:LYS:HD2	1.77	0.49
1:A:122:VAL:HG23	1:A:149:ILE:HG21	1.96	0.48
2:B:69:LYS:HG2	2:B:103:TYR:OH	2.14	0.48
1:C:57:VAL:HG11	1:C:174:LEU:HD22	1.97	0.47
2:B:98:MET:HE1	2:B:129:MET:HG2	1.97	0.46
2:B:61:ARG:CD	2:B:95:GLU:OE1	2.64	0.46
1:C:67:LEU:O	1:C:71:GLN:HG3	2.16	0.46
3:D:30:ARG:NH2	3:D:32:GLU:OE1	2.50	0.45
1:C:166:PHE:HA	1:C:169:ILE:HD12	1.98	0.45
1:A:76:ALA:O	1:A:79:ARG:HG2	2.17	0.45
1:A:7:VAL:N	6:S:135:HOH:O	2.49	0.45
1:A:137:LYS:HD3	1:A:137:LYS:HA	1.84	0.44
1:C:51:MET:HG2	1:C:56:LEU:HA	2.01	0.43

Continued on next page...

PRELIMINARY VALIDATION REPORT

Continued from previous page...

Atom-1	Atom-2	Interatomic distance (Å)	Clash overlap (Å)
2:B:85:LEU:HD11	2:B:97:VAL:HG13	2.00	0.43
1:C:89:ASP:HB3	1:C:95:THR:HG21	2.00	0.43
2:B:118:LYS:HE2	2:B:118:LYS:HB3	1.87	0.42
2:B:20:GLU:N	2:B:20:GLU:OE1	2.39	0.42
2:B:40:LYS:HE3	1:C:69:ARG:HA	2.00	0.42
1:C:152:PHE:HZ	1:C:168:THR:HG1	1.65	0.41
2:B:30:ARG:HH11	2:B:32:GLU:CB	2.33	0.41
3:D:118:LYS:O	3:D:121:THR:HG22	2.21	0.41
3:D:85:LEU:HD21	3:D:107:THR:HG22	2.02	0.41
1:C:41:ILE:HA	1:C:70:PHE:HB3	2.03	0.41
2:B:64:VAL:HG12	2:B:99:LEU:HD23	2.03	0.41
1:C:9:LEU:HD13	1:C:173:ALA:HB3	2.03	0.41
1:A:138:ARG:NH1	6:S:255:HOH:O	2.39	0.40
1:C:126:LYS:HG2	5:F:1401:GTP:C6	2.56	0.40
1:C:41:ILE:HG12	1:C:70:PHE:CD1	2.56	0.40
1:C:120:PHE:O	1:C:150:PRO:HD2	2.22	0.40

All (1) symmetry-related close contacts are listed below. The label for Atom-2 includes the symmetry operator and encoded unit-cell translations to be applied.

Atom-1	Atom-2	Interatomic distance (Å)	Clash overlap (Å)
1:A:49:GLU:OE2	6:S:45:HOH:O[4_545]	2.11	0.09

5.3 Torsion angles ⓘ

5.3.1 Protein backbone ⓘ

In the following table, the Percentiles column shows the percent Ramachandran outliers of the chain as a percentile score with respect to all X-ray entries followed by that with respect to entries of similar resolution.

The Analysed column shows the number of residues for which the backbone conformation was analysed, and the total number of residues.

Mol	Chain	Analysed	Favoured	Allowed	Outliers	Percentiles	
1	A	169/199 (85%)	164 (97%)	5 (3%)	0	100	100
1	C	169/199 (85%)	161 (95%)	8 (5%)	0	100	100
2	B	133/135 (98%)	132 (99%)	1 (1%)	0	100	100
3	D	133/135 (98%)	130 (98%)	3 (2%)	0	100	100

Continued on next page...

PRELIMINARY VALIDATION REPORT

Continued from previous page...

Mol	Chain	Analysed	Favoured	Allowed	Outliers	Percentiles	
All	All	604/668 (90%)	587 (97%)	17 (3%)	0	100	100

There are no Ramachandran outliers to report.

5.3.2 Protein sidechains ⓘ

In the following table, the Percentiles column shows the percent sidechain outliers of the chain as a percentile score with respect to all X-ray entries followed by that with respect to entries of similar resolution.

The Analysed column shows the number of residues for which the sidechain conformation was analysed, and the total number of residues.

Mol	Chain	Analysed	Rotameric	Outliers	Percentiles	
1	A	148/173 (86%)	147 (99%)	1 (1%)	85	89
1	C	148/173 (86%)	145 (98%)	3 (2%)	58	63
2	B	112/112 (100%)	106 (95%)	6 (5%)	24	21
3	D	111/111 (100%)	108 (97%)	3 (3%)	48	51
All	All	519/569 (91%)	506 (98%)	13 (2%)	50	55

All (13) residues with a non-rotameric sidechain are listed below:

Mol	Chain	Res	Type
1	A	31	LYS
2	B	14	ARG
2	B	24	LEU
2	B	43	SER
2	B	52	LEU
2	B	127	ARG
2	B	138	GLN
1	C	95	THR
1	C	137	LYS
1	C	140	GLN
3	D	14	ARG
3	D	69	LYS
3	D	95	GLU

Some sidechains can be flipped to improve hydrogen bonding and reduce clashes. All (1) such sidechains are listed below:

PRELIMINARY VALIDATION REPORT

Mol	Chain	Res	Type
2	B	65	GLN

5.3.3 RNA (i)

There are no RNA molecules in this entry.

5.4 Non-standard residues in protein, DNA, RNA chains (i)

There are no non-standard protein/DNA/RNA residues in this entry.

5.5 Carbohydrates (i)

There are no carbohydrates in this entry.

5.6 Ligand geometry (i)

Of 4 ligands modelled in this entry, 2 are monoatomic - leaving 2 for Mogul analysis.

In the following table, the Counts columns list the number of bonds (or angles) for which Mogul statistics could be retrieved, the number of bonds (or angles) that are observed in the model and the number of bonds (or angles) that are defined in the Chemical Component Dictionary. The Link column lists molecule types, if any, to which the group is linked. The Z score for a bond length (or angle) is the number of standard deviations the observed value is removed from the expected value. A bond length (or angle) with $|Z| > 2$ is considered an outlier worth inspection. RMSZ is the root-mean-square of all Z scores of the bond lengths (or angles).

Mol	Type	Chain	Res	Link	Bond lengths			Bond angles		
					Counts	RMSZ	$\#\|Z\| > 2$	Counts	RMSZ	$\#\|Z\| > 2$
5	GTP	E	1401	4	27,34,34	0.92	1 (3%)	29,54,54	1.87	6 (20%)
5	GTP	F	1401	4	27,34,34	0.94	1 (3%)	29,54,54	1.78	6 (20%)

In the following table, the Chirals column lists the number of chiral outliers, the number of chiral centers analysed, the number of these observed in the model and the number defined in the Chemical Component Dictionary. Similar counts are reported in the Torsion and Rings columns. '-' means no outliers of that kind were identified.

Mol	Type	Chain	Res	Link	Chirals	Torsions	Rings
5	GTP	E	1401	4	-	0/18/38/38	0/3/3/3
5	GTP	F	1401	4	-	0/18/38/38	0/3/3/3

All (2) bond length outliers are listed below:

PRELIMINARY VALIDATION REPORT

Mol	Chain	Res	Type	Atoms	Z	Observed(Å)	Ideal(Å)
5	E	1401	GTP	C6-N1	2.90	1.38	1.33
5	F	1401	GTP	C6-N1	3.07	1.38	1.33

All (12) bond angle outliers are listed below:

Mol	Chain	Res	Type	Atoms	Z	Observed(°)	Ideal(°)
5	F	1401	GTP	N3-C2-N1	-4.96	120.14	127.41
5	E	1401	GTP	N3-C2-N1	-4.82	120.34	127.41
5	E	1401	GTP	C5-C6-N1	-3.90	117.93	123.47
5	F	1401	GTP	C5-C6-N1	-3.00	119.20	123.47
5	E	1401	GTP	C4-C5-N7	-2.47	107.02	109.41
5	F	1401	GTP	PB-O3B-PG	-2.09	125.60	132.63
5	E	1401	GTP	PB-O3B-PG	-2.07	125.66	132.63
5	F	1401	GTP	PA-O3A-PB	-2.00	125.89	132.63
5	F	1401	GTP	C6-N1-C2	2.97	120.34	116.06
5	E	1401	GTP	C2-N3-C4	3.26	118.97	115.16
5	E	1401	GTP	C6-N1-C2	3.81	121.54	116.06
5	F	1401	GTP	C2-N3-C4	4.38	120.27	115.16

There are no chirality outliers.

There are no torsion outliers.

There are no ring outliers.

1 monomer is involved in 1 short contact:

Mol	Chain	Res	Type	Clashes	Symm-Clashes
5	F	1401	GTP	1	0

5.7 Other polymers (i)

There are no such residues in this entry.

5.8 Polymer linkage issues (i)

There are no chain breaks in this entry.

PRELIMINARY VALIDATION REPORT

6 Fit of model and data (i)

6.1 Protein, DNA and RNA chains (i)

In the following table, the column labelled '#RSRZ> 2' contains the number (and percentage) of RSRZ outliers, followed by percent RSRZ outliers for the chain as percentile scores relative to all X-ray entries and entries of similar resolution. The OWAB column contains the minimum, median, 95th percentile and maximum values of the occupancy-weighted average B-factor per residue. The column labelled 'Q< 0.9' lists the number of (and percentage) of residues with an average occupancy less than 0.9.

Mol	Chain	Analysed	<RSRZ>	#RSRZ>2			OWAB(Å^2)	Q<0.9
1	A	171/199 (85%)	-0.07	6 (3%)	44	51	16, 26, 41, 58	0
1	C	171/199 (85%)	1.08	34 (19%)	1	1	34, 55, 90, 116	0
2	B	135/135 (100%)	0.53	13 (9%)	8	10	24, 42, 73, 89	0
3	D	135/135 (100%)	0.31	8 (5%)	22	28	22, 35, 56, 65	0
All	All	612/668 (91%)	0.47	61 (9%)	7	9	16, 38, 74, 116	0

All (61) RSRZ outliers are listed below:

Mol	Chain	Res	Type	RSRZ
1	C	56	LEU	10.5
2	B	34	ILE	6.4
1	C	7	VAL	6.2
1	C	55	ARG	6.2
1	C	52	VAL	6.1
1	C	53	ASP	6.0
1	C	54	ASP	5.8
1	C	178	THR	5.3
1	C	51	MET	4.9
1	C	144	TYR	4.7
2	B	32	GLU	4.7
1	C	130	GLU	4.5
1	C	57	VAL	4.5
2	B	31	ASN	4.3
1	C	123	LEU	4.3
2	B	30	ARG	4.1
2	B	33	VAL	3.8
1	C	137	LYS	3.8
1	C	8	LEU	3.7
1	C	174	LEU	3.5
2	B	29	ALA	3.5

Continued on next page...

PRELIMINARY VALIDATION REPORT

Continued from previous page...

Mol	Chain	Res	Type	RSRZ
3	D	85	LEU	3.2
1	C	87	VAL	3.2
3	D	52	LEU	3.0
1	A	123	LEU	2.9
1	C	171	ARG	2.9
1	C	85	VAL	2.9
1	C	47	THR	2.8
1	C	140	GLN	2.8
2	B	138	GLN	2.8
1	A	86	LEU	2.7
1	C	131	ASN	2.7
3	D	56	CYS	2.7
3	D	125	GLU	2.7
3	D	84	PRO	2.6
1	C	148	ASN	2.6
1	A	87	VAL	2.6
1	C	31	LYS	2.4
1	C	133	GLN	2.4
1	C	32	LYS	2.4
1	A	85	VAL	2.4
1	C	152	PHE	2.3
2	B	19	GLU	2.3
2	B	132	ALA	2.3
1	A	178	THR	2.3
1	C	169	ILE	2.3
1	C	94	ASN	2.3
2	B	26	GLU	2.3
2	B	42	ARG	2.3
3	D	55	ALA	2.2
3	D	135	ARG	2.2
2	B	52	LEU	2.2
1	C	117	ASN	2.1
1	C	122	VAL	2.1
1	A	54	ASP	2.1
2	B	85	LEU	2.1
3	D	124	GLU	2.1
1	C	176	GLN	2.1
1	C	141	ALA	2.0
1	C	19	VAL	2.0
1	C	36	GLN	2.0

PRELIMINARY VALIDATION REPORT

6.2 Non-standard residues in protein, DNA, RNA chains (i)

There are no non-standard protein/DNA/RNA residues in this entry.

6.3 Carbohydrates (i)

There are no carbohydrates in this entry.

6.4 Ligands (i)

In the following table, the Atoms column lists the number of modelled atoms in the group and the number defined in the chemical component dictionary. The B-factors column lists the minimum, median, 95th percentile and maximum values of B factors of atoms in the group. The column labelled 'Q< 0.9' lists the number of atoms with occupancy less than 0.9.

Mol	Type	Chain	Res	Atoms	RSCC	RSR	B-factors($Å^2$)	Q<0.9
4	MG	F	1301	1/?	0.90	0.07	37,37,37,37	0
5	GTP	F	1401	32/?	0.97	0.10	28,44,50,51	0
4	MG	E	1301	1/?	0.98	0.08	18,18,18,18	0
5	GTP	E	1401	32/?	0.99	0.09	14,18,20,21	0

6.5 Other polymers (i)

There are no such residues in this entry.

PRELIMINARY VALIDATION REPORT
